# Velopharyngeal Anatomy and Speech Production in Cleft Palate Surrounding Primary Palatoplasty: Protocol for a Prospective Observational Pilot Study

**DOI:** 10.2196/97206

**Published:** 2026-07-10

**Authors:** Katelyn Kotlarek, Gregory Allen, Ilana Neuberger, Kate Bunton

**Affiliations:** 1 Division of Communication Disorders College of Health Sciences University of Wyoming Laramie, WY United States; 2 Children's Hospital Colorado Aurora, CO United States; 3 University of Colorado Anschutz Medical Campus Aurora, CO United States; 4 Department of Speech, Language, and Hearing Sciences College of Science University of Arizona Tucson, AZ United States

**Keywords:** cleft palate, velopharyngeal insufficiency, magnetic resonance imaging, soft palate, surgery

## Abstract

**Background:**

Children with cleft palate often experience impaired speech due to atypical velopharyngeal anatomy following palate repair surgery. While surgical repair aims to restore the function of the palate, more than one-third of cases result in continued velopharyngeal insufficiency (VPI) and require reoperation, which can result in negative psychosocial impacts and financial burden. Common surgical approaches for primary palatoplasty, including intravelar veloplasty and double-opposing Z-plasty, have similar reported rates of VPI. Furthermore, procedure selection occurs without any presurgical imaging and varies by operating surgeon. Surgical decisions are often based on intraoperative judgment rather than objective measures. Structural and functional variables have been associated with speech outcomes in this population, but these data exist independently of one another until the school-age years, failing to establish a direct connection between anatomy and functionality of the velopharynx in toddlers. The current clinical paradigm misses a critical window of opportunity for earlier diagnosis of VPI.

**Objective:**

The purpose of this study is to (1) establish which presurgical anatomical variables are predictive of surgical procedure selection based on perceptual assessment of intraoperative tension for palate repair and (2) determine which postsurgical anatomical features are associated with the greatest diversity in oral stop consonant production at 18 months in children with a repaired cleft palate.

**Methods:**

A total of 30 infants with cleft palate will be recruited prior to palate repair. Within 2 weeks of repair, participants will undergo nonsedated magnetic resonance imaging (MRI) of the velopharynx and produce a home-based speech recording. Participants will complete the same protocol after palate repair at 18 months of age. Data will be analyzed by independent raters following completion of a training protocol. Anatomical dimensions of the velopharynx will be extracted from the MRI scans. The number and diversity of oral stop consonants will be extracted from the speech recordings. Interrater and intrarater reliability will be assessed. Logistic regression will be used to evaluate which presurgical anatomical measurements are predictive of surgical procedure selection. Analysis of covariance will be used to examine whether postsurgical anatomical measurements are associated with diversity of oral stop consonant production.

**Results:**

This project was funded in August 2024. Data collection began in April 2025. As of April 4, 2026, a total of 8 participants had been enrolled, and data analysis had not begun. Results are anticipated in 2027.

**Conclusions:**

This study will use nonsedated MRI to investigate how velopharyngeal anatomy influences surgical decision-making and early speech outcomes. Such knowledge may be useful for presurgical planning and early monitoring of postsurgical speech outcomes in children with cleft palate.

**International Registered Report Identifier (IRRID):**

DERR1-10.2196/97206

## Introduction

### Background

Cleft palate is a common congenital anomaly in the United States, affecting roughly 6 per 10,000 live births. Cleft palate alone occurs in approximately 6 per 10,000 live births, whereas cleft lip with or without cleft palate occurs in approximately 10 per 10,000 live births [1]. A cleft palate can impact multiple aspects of a person’s life, including feeding, hearing, communication, dentition, and psychosocial health. Regarding speech, a combination of surgical and behavioral management is often undertaken to mitigate the long-term impact of cleft palate on a person’s ability to communicate.

Primary palate repair is typically performed at the ages of between 9 and 14 months in the United States [2]. Although age-based surgical protocols are common, practice varies due to individual differences in both the operating surgeon and patient-specific characteristics. Optimal timing remains debated, with earlier repair potentially supporting speech and velopharyngeal outcomes whereas later repair may reduce surgical complexity, facial growth restriction, and anesthesia-related risks [2-4]. Recent literature has increasingly supported earlier repair due to these potential speech benefits, although concerns regarding facial growth restriction and perioperative risks persist [5].

Anatomical restoration is the very premise for current surgical approaches to cleft palate repair. One goal is to create a tension-free closure of the cleft to improve healing and reduce the risk of reopening (ie, palatal fistula [6]). For clefts involving the soft palate, surgery should include reconstruction of the levator veli palatini (LVP) muscle. The LVP muscle, which functions to elevate and retract the soft palate, assists in the closure of the velopharyngeal port. Both intravelar veloplasty (IVV) and Furlow double-opposing Z-plasty (DOZ) are widely used techniques to reconstruct the LVP muscle [6]. IVV focuses on levator sling reconstruction with retropositioning and transverse reorientation of the muscles, whereas DOZ combines levator muscle repositioning with soft palate lengthening through opposing Z-plasty flaps. A systematic review by Timbang et al [7] found that outcomes following cleft palate repair vary by cleft type, with DOZ associated with lower rates of secondary velopharyngeal surgery compared with IVV. One way of reducing tension is to base the surgical plan on patient anatomy, where the surgeon determines procedure type based on the perceived tension of the palatal tissue in the operating room; as DOZ requires overlap of the LVP muscle bundles, it can produce a greater amount of tension on the incision site [7]. Beyond the surgeons’ informal assessment of tension intraoperatively, we do not know whether the child’s presurgical anatomy plays a role in their postrepair outcome, and there is no data-driven approach to choosing one surgical technique over another.

Some children still require additional surgical intervention due to remaining velopharyngeal insufficiency (VPI) after primary palatoplasty. VPI is an anatomical deficiency where the velopharyngeal port cannot adequately close to fully separate the oral and nasal cavities during speech and/or swallowing. VPI results in hypernasality, nasal air emission, and/or weak pressure consonants. Children with VPI are also at greater risk of compensatory articulation errors, which are abnormal, learned sound substitutions [8]. All these speech issues impact a person’s ability to communicate effectively. Rates of VPI range widely among hospitals, regions, and countries between 13% and 40% [9,10]. Inability of the initial palate repair surgery to address VPI places children at risk of long-term psychosocial challenges and increases the likelihood of additional costly interventions [11-13]. Even children with otherwise successful repairs often require speech therapy, with 34% to 68% exhibiting cleft speech characteristics [14-16].

Despite advances in surgical training and multidisciplinary cleft care, the incidence of speech disorders after repair has remained high for decades [17]. The timing and technique of primary palatoplasty have been shown to influence surgical outcomes [18]; however, these do not fully explain why some children continue to experience speech difficulties. Earlier repairs tend to result in reduced hypernasality and improved consonant production [14,19], but not all studies agree, with some reporting no clear age effect in secondary VPI surgery [3]. Recent prospective evidence further supports an association between timing and early speech sound development, particularly oral stop consonant production. A recent study of 113 infants with repaired cleft palate revealed that at least one oral stop was evident in the consonant inventory for 84% of children at 16 months, 71% produced 2 or more different stops, and only 7% produced all 6 stop consonants [20]. This represents a gap in the literature supporting early surgical and speech outcomes for children with cleft palate.

Cleft palate repair aims to restore functional separation of the oral and nasal cavities to support speech development through reconstruction of the soft palate. Common approaches include IVV to reposition the levator musculature and DOZ, which uses opposing Z-shaped flaps to both lengthen the soft palate and improve muscular alignment; both are typically performed in conjunction with primary palatoplasty techniques such as the 2-flap or similar layered closure approaches. Both techniques are widely used in clinical practice, but comparative studies have not established a consistently superior approach, with outcomes varying across patient populations and study designs [7,21].

### Innovation in Magnetic Resonance Imaging–Based Assessment

The clinical gold standard for assessing VPI is perceptual evaluation and nasopharyngoscopy, which is commonly not possible in children under 3 years of age due to reduced speech complexity and compliance [22,23]. Magnetic resonance imaging (MRI) offers a noninvasive method to visualize palatal musculature in vivo and has been successfully applied to study cleft palate in both adult [24-28] and pediatric populations [29-31]. Nonsedated MRI protocols, when combined with behavioral preparation, improve compliance and procedural success in infants, enabling assessments both before and after palate repair. This provides the opportunity to connect patient-specific anatomical features with early speech outcomes. MRI-based assessment in infants has the potential to help identify VPI risk much earlier than the current clinical timeline allows. Additionally, using MRI to quantify surgical outcomes moves beyond subjective perceptual assessments, which are prone to bias, and supports data-driven, individualized surgical planning and monitoring. Nonsedated MRI also addresses the limitations of conventional imaging methods such as sedation-related cognitive risks, radiation exposure, and invasiveness.

MRI of the velopharynx is becoming increasingly accepted to assess older children with VPI [32,33], but it has not yet been applied clinically to children under 4 years of age. Most prior research has focused on VPI rates or surgical technique comparisons rather than exploring the mechanisms behind these speech deficits [17]. Understanding velopharyngeal development in typical populations reveals substantial variability in timing and sound-specific closure patterns [34], highlighting the need for studies focusing on these underlying mechanisms. This study aims to fill these gaps by using MRI to connect individual anatomy to early speech development in children with cleft palate.

### Aims of This Study

In summary, recent studies quantifying the velopharynx in children have generally examined anatomy and physiology in isolation [35], and many have relied on cross-sectional designs [15,32]. Few have tracked both anatomical and functional measures at 2 time points within the same individual. Multidimensional data before and after palatoplasty are crucial for understanding how palate repair influences both velopharyngeal structure and function. These individualized assessments are key for translating basic science findings into patient-specific surgical planning and targeted interventions. This protocol aims to take an essential step toward precision medicine in infants with cleft palate.

This study combines nonsedated MRI–based anatomical assessment with functional speech measurements in the same infants before and after palatoplasty. By linking patient-specific anatomy before and after palate repair to early speech outcomes, we aim to establish data-driven criteria for individualized surgical planning and targeted speech monitoring. This approach addresses a critical gap by providing quantitative, objective measures of surgical success and velopharyngeal development, moving beyond subjective assessment. Ultimately, these findings may lead to work that reduces persistent speech deficits, prevents multiple surgeries, and lessens the associated psychosocial and financial burden of cleft palate. Specifically, this study has two primary aims:

1. To establish which presurgical anatomical variables are predictive of surgical procedure selection based on perceptual assessment of intraoperative tension for palate repair (hypothesis: preoperative cleft width and LVP muscle length are predictive of surgical procedure selection based on intraoperative tension of palatal tissue)
2. To determine which postsurgical anatomical features are associated with the greatest diversity in oral stop consonant production at 18 months in children with repaired cleft palate (hypothesis: a longer velum, greater effective velopharyngeal ratio, and continuity of the LVP muscle are associated with greater diversity of oral stop consonants by 18 months but not with procedure type)

## Methods

This observational pilot study will examine the relationships among velopharyngeal anatomy, surgical procedure choice, and early speech outcomes in infants with cleft palate.

### Ethical Considerations

The study was approved by the Colorado Multiple Institutional Review Board (protocol number 22-1135). A reliance agreement is in place with the University of Wyoming Institutional Review Board. Written informed consent will be obtained from all caregivers prior to enrollment. Additional consent provisions are in place for continued data collection and monitoring of the medical chart up to 18 months of age and the use of participant data in ancillary studies; participants may opt out of follow-up at any time.

### Participants and Recruitment

A total of 30 participants will be recruited from the Children’s Hospital Colorado Cleft Lip and Palate Program (Aurora, Colorado), with an initial enrollment of 39 to account for an anticipated 30% attrition due to MRI motion artifacts, refusal, or missed visits. This sample size was selected based on the pilot nature of this work and use of a single recruitment site; furthermore, the sample size is consistent with prior prospective studies using nonsedated MRI of the velopharynx in children [33,36,37]. This study is considered exploratory to evaluate the relationship among anatomy, surgical selection, and early speech outcomes, as well as to support future larger-scale, multicenter studies. The recruitment site receives 50 to ⁠60 new referrals each year, providing ample opportunities to reach the target sample size over the 3-year funding period. Retention strategies will be used to reduce dropout, including regular personalized reminders, flexible scheduling, and incentives. Participants must be from English-speaking families and have a diagnosis of cleft palate that has not yet been repaired. Infants diagnosed with syndromes prior to 18 months (as reported in their medical chart), those ineligible for MRI (eg, metal implants or severe airway anomalies), or those with other contraindications to standard surgical care will be excluded. Those scheduled for primary palatoplasty after 15 months of age will also be excluded from this analysis.

### Surgical Considerations

Participants will undergo primary palatoplasty within a typical early palate repair window prior to 15 months [38]. Two standard surgical approaches will be evaluated: IVV and Furlow DOZ. These 2 procedures are commonly used for primary palatoplasty at the study site. Due to the observational nature of this study, no standardization or changes to the surgeons’ procedures will take place; however, individual variability will be accounted for by recording the operating surgeon of each participant and controlling for any significant differences between surgeons in the analysis. Both procedures aim to restore the LVP “sling” to its intended anatomical position in the posterior velum but differ in technique. IVV reorients the muscle bundles end to end without overlap and closes the mucosa with a straight-line suture. DOZ repositions the LVP muscle via opposing Z-shaped incisions, lengthening the velum and creating overlap of the muscle bundles. All surgeries will be performed at a single institution by 1 of 5 operating surgeons. Procedure selection and the use of tissue adjuncts will be determined intraoperatively based on the surgeon’s clinical expertise, which may include a perceptual assessment of the amount of palatal tissue and/or tension upon closure of the cleft defect; no randomization will be performed. Tissue adjuncts, in this context, refer to flaps or grafts added to the palate from an alternate location in the mouth (eg, buccal fat pad flap or buccal myomucosal flap).

### Study Design and Data Collection

**Figure 1:**
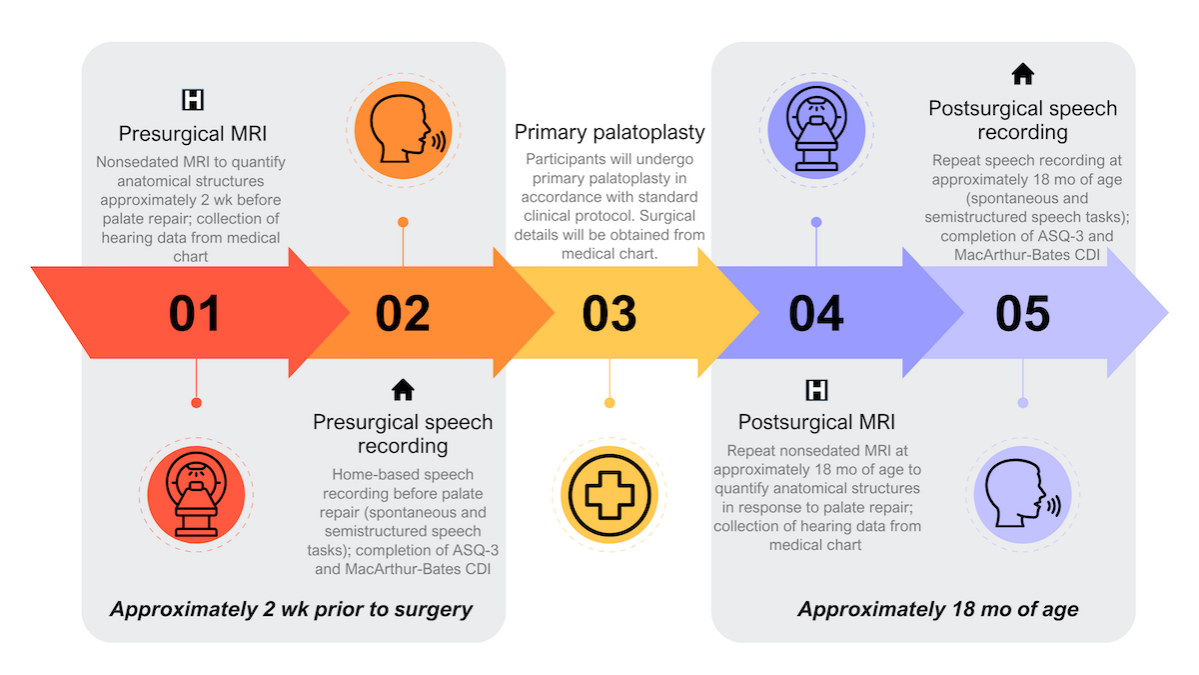
Participant-level timeline for multimodal data collection throughout the study. ASQ-3: Ages and Stages Questionnaire, Third Edition; CDI: Communicative Development Inventories; MRI: magnetic resonance imaging.

As part of the study protocol, MRI scans will be acquired presurgically (approximately 10-12 months depending on surgical timing) and at approximately 18 months of age. These time points were selected to reflect changes in both speech production and anatomy before and after surgery. The first time point, which will occur within 2 weeks prior to the scheduled primary palatoplasty, is relevant for imaging used in presurgical planning. While the timing of the presurgical MRI relative to surgery may vary based on clinical scheduling, all MRI data will be collected solely for research purposes and will not be used to guide surgical decision‑making. Surgeons will not be provided with study MRI findings when determining the surgical approach. At each visit, infants will undergo a nonsedated MRI of the velopharynx using a “feed and wrap” protocol [39], with age- and size-appropriate hearing protection and head stabilization cushions. Fast 2D T2-weighted turbo spin echo scans will be acquired first to minimize motion artifacts, followed by high-resolution 3D anatomical scans covering the oropharyngeal anatomy. MRI datasets will be processed and analyzed using Amira Software (Thermo Fisher Scientific), with custom resampling to visualize the entire LVP muscle from origin to insertion via an oblique-coronal plane. Details on type, location, and timing of data collection are outlined in Figure 1.

At both time points, caregivers will also complete an at-home speech recording using a LENA digital language processor (LENA Foundation) worn in a custom vest, along with a structured book reading task to capture 2 to 4 hours of the child’s vocalizations and target words. Audio files will be analyzed using modified naturalistic listening in real time (NLRT) [20,40,41]. Per the NLRT protocol, consonants will be included in the analysis only if they are produced at least twice across the sampled segments. Reflexive vocalizations will be excluded, whereas meaningful productions, including whispered or sung sounds, will be included. The primary outcome measure is consonant inventory size, defined as the total number of distinct consonants meeting the inclusion criterion across the full analyzed sample.

At both time points, caregivers will complete an intake form, the Ages and Stages Questionnaire, Third Edition [42], and the MacArthur-Bates Communicative Development Inventories [43]. Hearing-related data will be taken from the participants’ medical charts from birth up to the 18-month time point to characterize developmental, communicative, and middle-ear status alongside MRI and speech data (see Textbox 1 for a summary of participant data collected at each visit). Hearing data may include tympanometry, otitis media, and newborn hearing screening results, as available. Standardized procedures for MRI acquisition and speech data collection will ensure reliability and minimize the burden on participants and caregivers. These time points are supported by prior research demonstrating that velopharyngeal closure and early speech production are measurable in infants and toddlers with and without cleft palate. Specifically, velopharyngeal closure is largely complete by 19 months [34], some children with repaired cleft palate achieve consistent closure for stop consonants by 14 months [35], and modified NLRT provides reliable assessment of early consonant production as early as 16 months [40].

**Participant data collected after enrollment and updated for each visit.**

**Demographic information**

- Exact age (measured in years to 2 decimal places)
- Sex (male or female)
- Race
- Height (measured in inches)
- Weight (measured in pounds)
- Head circumference (measured in inches)
- Cleft type

**Hearing information**

- Hearing status (tympanometry; pass or fail and date)
- Ear infections (yes or no and how many if yes)
- Other hearing information

**Speech information**

- Speech therapy (yes or no; if yes, when and what was targeted)
- Ages and Stages Questionnaire, Third Edition (complete form)
- MacArthur-Bates Communicative Development Inventories (complete form)

**Surgical information**

- Palate repair (intravelar veloplasty or double-opposing Z-plasty; date; whether there was adjunct tissue; and, if yes, whether it was buccal fat flaps or buccal myomucosal flaps)
- Pressure-equalizing tubes (yes or no and the date if yes)

Two independent raters will measure the speech and/or MRI data. The primary rater will be blind to the participant demographics to reduce bias. This will be achieved through controlling access to participant demographics and deidentifying the raw data for analysis.

### Evaluation Outcomes

The primary outcomes correspond to the 2 study aims. For aim 1, the primary outcome is the type of surgical procedure performed (IVV or DOZ with or without adjunct tissue use) predicted by preoperative anatomical variables, namely, cleft width and LVP muscle length. For aim 2, the primary outcome is the diversity of oral stop consonants produced at 18 months, serving as a functional measure of velopharyngeal competence. Secondary outcomes include additional anatomical measures (effective velar length, effective velopharyngeal ratio, and LVP muscle continuity) and developmental and language scores. Anatomical variable definitions are outlined in Table 1. Exploratory tertiary outcomes will examine modifiers of anatomy and speech, including sex, demographic characteristics, tympanometry results, and other developmental assessments. Missing data will be imputed (when appropriate) or addressed using pairwise deletion. Additionally, data may be analyzed cross-sectionally to meet the study aims.

**Table 1:** Anatomical variables and corresponding definitions as obtained from magnetic resonance imaging for aims 1 and 2.

Variable	Definition
Cleft width	Distance between the lateral margins of the cleft at the widest point
Effective VP^a^ ratio	Effective velar length/pharyngeal depth
Effective velar length	Length of the velum from the posterior hard palate to the LVP^b^ muscle
LVP muscle angle of origin	Interior angle created by the lines drawn to measure distance between the origin points and LVP muscle length
LVP muscle continuity	Whether the LVP muscle is continuous through the midline (yes or no)
LVP muscle length	Length of the LVP muscle along one side from origin to insertion
Origin-origin distance	Distance between the points of origin of the LVP muscle
Pharyngeal depth	Distance from the posterior hard palate to the posterior pharyngeal wall at the palatal plane
VP ratio	Total velar length/pharyngeal depth
Velar insertion distance	Distance between the locations where the bilateral LVP muscle bundles insert into the body of the velum (ie, repaired velar width)
Velar length	Length of the velum from the posterior hard palate to the tip of the uvula
Velar thickness	Distance from the velar knee to the velar dimple

^a^VP: velopharyngeal.

^b^LVP: levator veli palatini.

Due to the observational nature of this study, there are minimal and foreseeable risks, primarily related to potential anxiety or fear associated with the MRI procedure. No sedation will be used, and all established MRI safety screening and monitoring procedures of the clinical site will be followed [44]. All events will be documented and managed according to institutional safety protocols. Caregiver questionnaires and clinical data will be entered into a secure REDCap (Research Electronic Data Capture; Vanderbilt University) database [45] with routine quality checks to maintain accuracy and completeness. Study data will be deidentified and securely stored on institutional servers. Access to study data will be limited to authorized research staff. After study completion, participants will continue standard clinical follow-up through the craniofacial team; no additional interventions beyond standard care are required.

### Statistical Analysis

All participants with data at the relevant time points will be included in each analysis. The primary outcome will be evaluated using binomial logistic regression to determine how preoperative anatomical variables predict surgical procedure selection, controlling for sex, race, and cleft type. Principal component analysis may be applied to reduce dimensionality among anatomical predictors. The secondary outcome will be assessed using a 2-way analysis of covariance examining the effects of surgical type and number of oral stops on postsurgical anatomical measures, controlling for sex, cleft type, and developmental and language scores.

To ensure scientific rigor, intrarater and interrater reliability will be calculated for both MRI and speech measures. Independent raters will measure MRI scans and consonant productions following completion of a structured reliability training protocol to enhance measurement accuracy and precision. This training will consist of a guided review of the velopharyngeal MRI and speech literature, a detailed study of relevant cleft MRI anatomy and speech characteristics, and repeated practice measurements of both typical children and those with cleft palate until a predetermined level of agreement is reached. Each measurement training cycle will be followed by discussion-based debriefing and troubleshooting. The primary rater will measure all samples, whereas a second rater will measure a randomly selected 10% of the samples to calculate interrater reliability. The primary rater will rerate a random selection of 10% of the samples to calculate intrarater reliability.

## Results

This study was funded in August 2024 by the National Institute on Deafness and Other Communication Disorders (R21DC021799). Recruitment and data collection for this study began in April 2025 and are currently underway. As of April 4, 2026, a total of 8 participants have been enrolled, and data analysis has not begun. It is anticipated that enrollment will be complete and final results will be disseminated by the end of 2027 following completion of data analysis.

## Discussion

### Expected Findings

This pilot study will explore the relationship between patient-specific anatomy before and after palate repair and early speech outcomes. Modern attempts to report quantitative measures of the velopharynx in infants have investigated its anatomy [30,46,47] and physiology [20,34,35,48] independently of one another and have commonly used a cross-sectional study design rather than comparing these variables within the same individual over time. Multidimensional data that encompass measures of both anatomy and physiology within the same individual over time are essential for examining how primary palatoplasty affects velopharyngeal form and function at the level of the individual patient. Individualized presurgical planning and responses to surgical treatment are essential for translating basic science findings into valuable patient-specific intervention protocols. This proposal has been designed to take this important step to facilitate precision medicine in this population. Preliminary, data-driven criteria for individualized surgical planning and targeted speech monitoring will be established as a result of this work. Pending a larger, multisite investigation, this work may have future implications to improve and individualize surgical interventions for primary palatoplasty.

**Figure 2:**
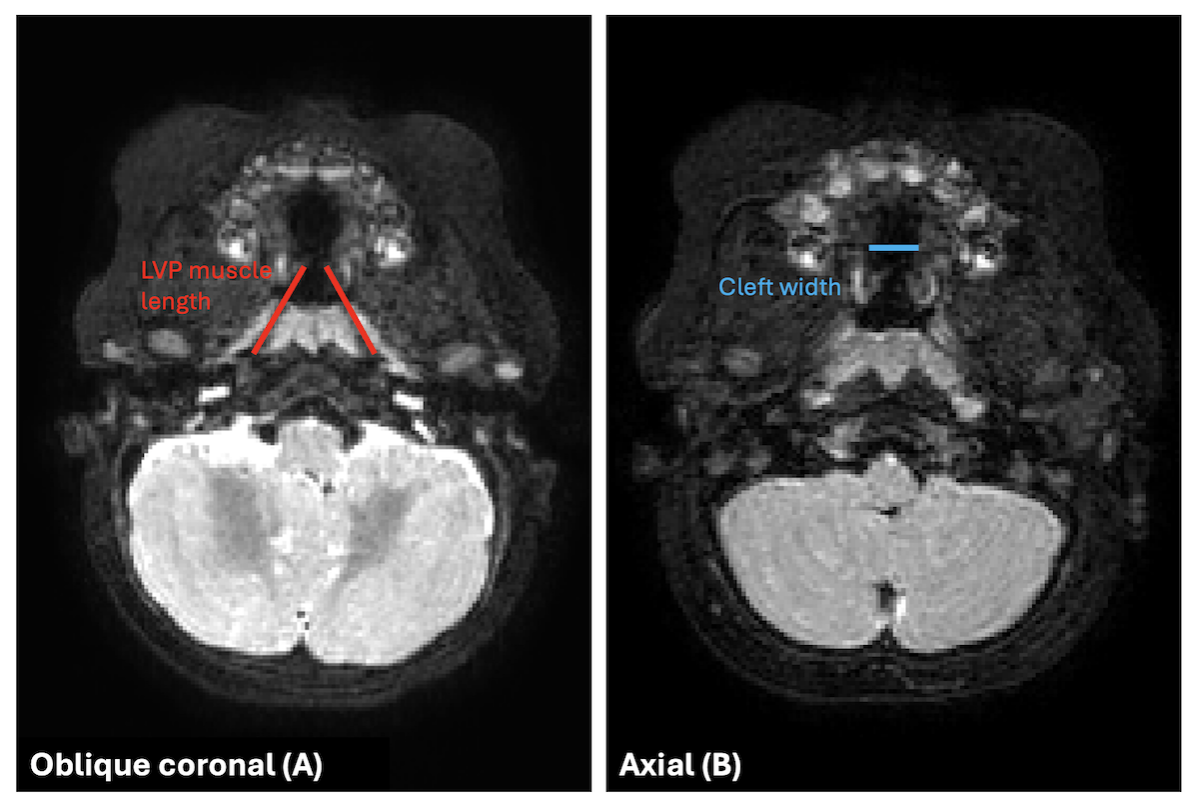
Presurgical magnetic resonance image of an infant labeled with cleft palate with cleft width (A) and levator veli palatini (LVP) muscle length (B).

Aim 1 will determine which anatomical variables from those used in previous investigations [30,46] can predict the type of surgery received, quantifying the surgical decision-making process objectively using MRI and reducing reliance on surgeon experience to make this decision. We hypothesize that certain anatomical parameters, namely, palatal width and LVP muscle length (Figure 2), may be predictive of surgical procedure selection. If this is the case, this study will provide a quantified, anatomical basis for individualized surgical interventions to support successful palatoplasty and potentially reduce VPI. Alternatively, if there is no anatomical link between patient-specific, presurgical anatomy and surgical decision-making, a more complex, multidimensional analysis of surgical decision-making factors is warranted to improve outcomes of primary palatoplasty and subsequent rates of VPI.

**Figure 3:**
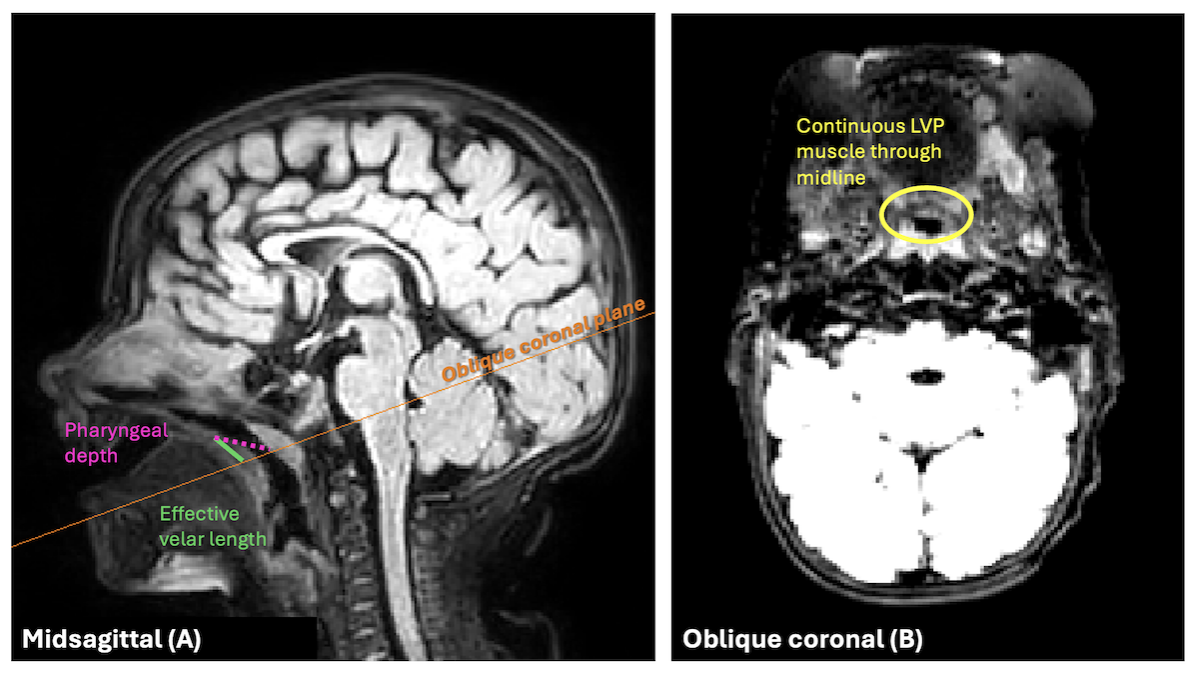
Magnetic resonance image of a typically developing toddler demonstrating the anatomy of interest when considering the risk of velopharyngeal insufficiency. LVP: levator veli palatini.

Through aim 2, we will compare velopharyngeal function for speech among infants with repaired cleft palate following different repair techniques using both perceptual (oral stop production) and objective (anatomical) measures of the velopharynx [29,31]. Given that surgery is an anatomical solution to a physiological concern (aberrant speech), it is essential to establish a link between anatomy and physiology in infants and toddlers to facilitate early diagnosis and remediation of VPI in this population. We further hypothesize that anatomical parameters such as a longer velum, greater effective velopharyngeal ratio, and continuity of the LVP muscle (Figure 3) may be associated with greater diversity of oral stop consonants by 18 months. If this hypothesis is accurate, postoperative anatomy of children with repaired cleft palate could be used as a complement for speech production when it comes to considering secondary surgical intervention. Identifying the relationship between postoperative anatomy and velopharyngeal function in toddlers offers a critical opportunity to intervene earlier and improve speech outcomes in this population. These multidimensional data may provide additional confidence in referring for secondary surgical intervention at an earlier age, when speech production is more limited in clinical visits.

It is well established that anatomical dimensions have a direct impact on adequate function of the velopharyngeal mechanism for typical speech production. This research will yield promising patient-specific criteria for surgical planning and the success of cleft repair, facilitating earlier and potentially reduced diagnosis of VPI by translating a highly innovative area of research to infants and improving the clinical feasibility of these procedures for VPI diagnosis. We will track participants beyond the 6-month period of this study through their annual cleft team visits to document which children are clinically diagnosed with VPI, measuring the sensitivity and specificity of MRI at 18 months compared to the current clinical paradigm. By applying anatomical findings associated with VPI in older children to infants and toddlers, we may be able to identify VPI risk 3 years earlier than the current clinical paradigm allows. Furthermore, this research addresses a critical gap by assessing surgical outcomes quantitatively rather than perceptually, thus reducing subjectivity and bias. Results may improve the clinical utility of MRI in the surgical planning and monitoring of individuals with cleft palate. These foundational findings may provide support for a forthcoming clinical trial to prospectively match patients with surgical techniques and monitor their outcomes.

Sedation of children for MRI is the typical standard of care worldwide, especially in children under 8 years of age. There is, however, a link between increased anesthetic events in childhood and suboptimal cognitive development [49]. Current imaging methods for children with VPI either are invasive (eg, nasendoscopy) or expose them to ionizing radiation (eg, videofluoroscopy [22,50]). Additionally, children below the age of 3 years are not typically candidates for either procedure due to compliance [22,23]. Nonsedated MRI of the velopharynx avoids these common imaging pitfalls while yielding better rates of patient acceptance and success and greater cost efficiency [50].

Findings will be disseminated through peer-reviewed publications and conference presentations. Any modifications to this protocol will be published along with the findings.

### Strengths and Limitations

This study has several strengths. A key strength is the use of nonsedated MRI to capture detailed velopharyngeal muscle anatomy in infants. This study is the first to link presurgical anatomy, surgical procedure selection, and early speech outcomes within the same cohort. The 2 time points in this study, combining structural MRI and home-based speech recordings, provide comprehensive, patient-specific data that are easily repeatable and not subject to perceptual bias.

This study also has some limitations. These limitations, however, are appropriate for the pilot nature of this work. The relatively small sample size and recruitment from a single hospital with multiple surgeons may reduce the ability to detect subtle effects and limit the broader applicability of the findings. Following the results of this pilot study, it is anticipated that these methods will be applied to a multisite study and larger population of children with cleft palate.

## Data Availability

Data sharing is not applicable to this paper as no datasets were generated or analyzed during this study.

## Abbreviations

DOZ: double-opposing Z-plasty
IVV: intravelar veloplasty
LVP: levator veli palatini
MRI: magnetic resonance imaging
NLRT: naturalistic listening in real time
REDCap: Research Electronic Data Capture
VPI: velopharyngeal insufficiency
